# The Paradox of the Mediterranean Diet in Pediatric Age during the COVID-19 Pandemic

**DOI:** 10.3390/nu14030705

**Published:** 2022-02-08

**Authors:** Elvira Verduci, Giulia Fiore, Elisabetta Di Profio, Gian Vincenzo Zuccotti

**Affiliations:** 1 Pediatric Department, “Vittore Buzzi” Children’s Hospital, 20154 Milan, Italy; giulia.fiore@unimi.it (G.F.); elisabetta.diprofio@unimi.it (E.D.P.); gianvincenzo.zuccotti@unimi.it (G.V.Z.); 2 Department of Health Sciences, University of Milan, 20142 Milan, Italy; 3 Department of Health, Animal Science and Food Safety, University of Milan, 20133 Milan, Italy

The outbreak of the COVID-19 pandemic, whose causative agent is Severe Acute Respiratory Syndrome Coronavirus 2, has caused a global crisis that has had a major impact on the health of the global population. However, the adverse effects of SARS-CoV-2 are not limited to the related infectious disease, as the pandemic has caused additional indirect health implications. Due to the COVID-19 pandemic, most countries have been forced to adopt containment and restriction measures to control the spread of the virus. These, in turn, have imposed changes in the lifestyle behaviors of adults and children worldwide, with potentially negative health consequences [1].

A greater rate of obesity and unfavorable eating habits has been documented during the COVID-19 pandemic in the healthy pediatric population. Considering the dramatic rates of pediatric obesity that already exist in Mediterranean countries [2], a number of studies have been published investigating the habits of children and adolescents during the period of isolation. For instance, in Greek children aged 2-to-18 years, the COV-EAT cross-sectional study showed alterations in eating behaviors during lockdown. In particular, increases in the intake of fruits, vegetables, dairy products, and pasta was shown along with a rise in the total consumption of sweets and total snacks. The latter, together with a decrease in physical activity, has been significantly associated with children’s and adolescents’ body weight excess. Thus, 35% of parents reported an increase in body weight in their children [3]. Additionally, a cross-sectional survey conducted among Spanish adolescents showed similar results. During confinement, there was an increase in unhealthy eating behaviors, such as more irregular patterns of meal distribution (39.9%) or an increase in snacking between meals (56.4%) [4]. In Italy, a survey carried out from the 5th to the 24th of April 2020 showed that a higher BMI, as well as a lower age, were associated with an increase in junk food consumption (packaged sweets and baked products, sweet beverages, savory snacks, and dressing sauces) (*p* = 0.005 and *p* < 0.001). Moreover, by administrating the validated Mediterranean diet adherence screener (MEDAS), the population group aged 18–30 years was shown to have a higher adherence to the Mediterranean diet (higher MEDAS score) when compared to the younger group aged 12–17 years (*p* < 0.001) [5].

Moreover, a cross-sectional study showed that lockdown worsened the eating habits of overweight and obese Italian children, and 67.2% of subjects had increased their consumption of homemade desserts, bread, pasta, and pizza [6]. Similarly, a longitudinal observational study documented negative changes in lifestyle, including diet (the intakes of potato chips, red meat, and sugary drinks all increased), physical activity, and sleep duration among obese Italian children during 3 weeks of national lockdown.

These findings suggest that COVID-19 restrictions have a consistent negative impact on the lifestyle behaviors of children living in the Mediterranean Area. Therefore, the term “covibesity” has been introduced to portray the aggravation in obesity rates due to the lockdown imposed during the pandemic [7]. Moreover, as recently discussed by the European Society for Clinical Nutrition and Metabolism (ESPEN), obesity could dangerously impact the severity of COVID-19 and has emerged as one of the most prominent risk factors increasing the disease mortality, which has also been found in children [8,9,10].

These data showed that the paradox that existed even before the pandemic (i.e., high rates of overweight and obesity despite having access to a dietary pattern recognized as one of the healthiest) has been greatly exacerbated, highlighting the need to closely monitor eating behaviors to avoid the development of unhealthy eating habits and prevent obesity. Informational tools and guidelines have been released as preventative strategies at the national level to spread healthy choices and guide people to get involved in the process of preparing food at home as an alternative to consuming high levels of processed foods [11].

Primary care has a central role in the provision of support by adopting initiatives and technologies to manage children at risk of obesity or already obese. For instance, the use of telehealth technology is a modern approach that is useful for the delivery of health care services by health care professionals, where distance is a critical factor. Telehealth interventions have been shown to improve access to treatment and screening rates of pediatric obesity over the last years [12]. The use of telemedicine has also had beneficial effects in obese pediatric patients and has been shown to stabilize or decrease BMI z-scores, increase physical activity, and improve eating habits [13,14]. In pediatric obesity, nutrition education for the whole family is one of the first treatments that should be implemented. Telemedicine visits are a great opportunity to get the whole family involved even under COVID-19 confinement. Using a computer, or other digital support, educational material can be shared and explained during the visit to make the patient and all stakeholders more active. Another potential benefit of telehealth nutrition care is that it increases access to medical nutrition therapy (MNT) by removing barriers related to limited finances, travel, time, and physical function, especially during the COVID-19 pandemic [15]. Teleconsultation should address not only nutritional support and weight control, but also increased physical activity and reduced sedentary behavior via the remote provision of exercise training programs [16]. Thus, an integrated care model for childhood obesity combining telehealth and face-to-face visits has recently been proposed. However, a few inherent limitations of this method do subsist, such as the measurement of auxological parameters and the evaluation of obesity-related complications [16].

As future perspectives, digital technology access, social and linguistic differences, and privacy security must be overcome to realize the full potential of telehealth for weight management among children and adolescents [17].

Overall, computer-based monitoring tools should be promoted within clinical facilities and hospitals for the prevention and treatment of childhood obesity. However, several challenges must be addressed, including the spread and the access to effective digital-care technology.

The development of comprehensive strategies is needed, and such measures might include specific intervention strategies that need to be adopted during confinement, the implementation of telemedicine with lifestyle and exercise programs, and supplemental guidance to families to maintain healthy lifestyle choices under COVID-19 restrictions.
